# Compartment Syndrome of the Lower Limb in Adults and Children and Effective Surgical Intervention and Post-surgical Therapies: A Narrative Review

**DOI:** 10.7759/cureus.63034

**Published:** 2024-06-24

**Authors:** Mikayla Hobbs, Hira T Rahman, Rhea Raj, Kesava Mandalaneni, Sudhakar Pemminati, Vasavi R Gorantla

**Affiliations:** 1 School of Medicine, St. George's University School of Medicine, St. George's, GRD; 2 Department of Neuroscience, St. George's University School of Medicine, St. George's, GRD; 3 Department of Pharmacology, California Health Sciences University College of Osteopathic Medicine, Clovis, USA; 4 Department of Biomedical Sciences, West Virginia School of Osteopathic Medicine, Lewisburg, USA

**Keywords:** pediatric compartment syndrome, decompressive fasciotomy, lower extremity trauma, acute compartment syndrome of leg, compartment syndrome leg

## Abstract

Compartment syndrome (CS) can be defined as an acutely painful condition that occurs due to increased pressure within a compartment, resulting in reduced blood flow and oxygen to nerves and muscles within the limb. It is considered a surgical emergency, and a delayed diagnosis may result in ischemia and eventual necrosis of the limb. The majority of cases in adults are associated with high-energy trauma, more specifically, long bone fractures of the lower limb, while supracondylar fractures of the humerus are highly associated with CS in pediatric patients. CS may also develop gradually as a result of prolonged and ongoing physical activity such as running. In this narrative review, we discuss the anatomy, pathophysiology, methods of diagnosis, and effective management of CS in adults and children.

## Introduction and background

Compartment syndrome (CS) is a medical condition characterized by increased pressure within an enclosed space (compartment) in the body. The elevated pressure within the compartment restricts the flow of blood to muscles, nerves, and other structures within that area, resulting in ischemia and potentially irreversible damage if not promptly treated. CS can be either acute or chronic. Acute CS (ACS) often results from trauma, fractures, crush injuries, or following a surgical procedure. It results in rapid swelling and an increase in pressure within the affected compartment. Seventy-five percent of cases of ACS are associated with high-energy trauma, more specifically, long bone fractures of the lower limb [1]. Tibial fractures in both adults and children have been associated as the biggest risk factor for developing ACS ranging from 1% to 9% [2]. A study also identified patient age, weight, fracture type, and mechanism of injury as other significant risk factors associated with the development of ACS. A multivariable logistic regression analysis confirmed that both being 14 years of age and older and the mechanism of injury were statistically significant risk factors and the best predictors for developing this condition [2]. Other etiologies include soft tissue injuries, burns, vascular injuries and crush injuries, and bleeding disorders such as hemophilia, thrombosis, and infections [1]. Pediatric patients present similarly in that tibial fractures are highly associated with CS, followed by supracondylar fractures of the humerus and then radial and ulnar fractures [1,3]. CS of the lower limb may result in the compression of the extensor muscles of the toes, the tibialis anterior muscle, the deep peroneal nerve, and the tibial artery [1]. This can all lead to detrimental and irreversible effects but can be mitigated with prompt diagnosis and fast time to surgical intervention.

ACS most commonly occurs in young men, which is believed to be because of a larger intracompartmental muscle mass and a higher likelihood of being involved in high-trauma situations [1]. The incidence of ACS is 7.3 per 100,000 in adult males, while it is 0.7 per 100,000 in adult females. Trauma-causing tibial shaft fractures have an incidence rate of 1-10% [1]. In children younger than 10, however, the etiology of CS is typically due to a vascular injury or infection, while in those older than 14 years, the etiology is usually due to trauma or surgical positioning. Similar to adults, male children are also more likely to develop CS compared to female children due to a higher rate of being involved in highly traumatic situations than their female counterparts [3].

ACS occurs within hours of trauma, and a swift diagnosis is vital to prevent irreversible harm. A thorough history and physical exam elicit a patient to express pain out of proportion to the injury or tense skin in the compartment affected. In the early stages, pain occurs with passive stretching, burning, and aching [1]. Other symptoms may include rapid swelling and an increase in pressure within the affected compartment with associated severe pain. Additional symptoms such as pallor, paresthesia, paralysis, and pulselessness may also be seen [1]. The evaluation of clinical symptoms can often be nonspecific because pain may be an unreliable indicator of impending harm. It would be logical to recognize that patients presenting as a result of trauma would experience severe pain. However, pain out of proportion following fracture stabilization and mild responses to analgesia would be alarming symptoms that physicians should be aware of. In cases of CS involving nerve damage, pain may not even be present. To make matters worse, if a patient is in a coma or on high-dose analgesia, clinical symptoms such as pain are likely to go unrecognized [4]. Diagnosis in a child becomes even more difficult because children often are unable to express their concerns. Pain could be exhibited in the forms of agitation and restlessness.

Chronic, or exertional, CS (CECS) on the other hand, typically develops gradually as a result of physical activity, usually in the lower limbs. It most commonly occurs in the anterior, lateral, and deep posterior compartments with symptoms presenting bilaterally 85%-95% of the time [5]. The incidence is most prevalent in running athletes; however, CECS can occur in any athlete [6]. Pediatric patients presenting with CECS are most commonly female runners [6]. One report presented an exceptionally rare case of CECS in a pediatric competitive figure skater [7]. Symptoms commonly arise during exercise and include pain, cramping, or aching that subsides with rest. Exercise can result in an increase in muscle size by up to 20%, leading to increased tissue pressure and compromise of microcirculation with subsequent tissue dysfunction and injury within a relatively unyielding muscle compartment [8]. A study by Hurschler et al. suggested that there are also changes in the mechanical properties of the fascia. They reported that the fascia of patients with CECS is thicker and stiffer than those of unaffected individuals [9]. The diagnosis of CECS is clinical and is usually delayed, with an average of 22 months between symptom onset and initiation of treatment [10].

## Review

Anatomy

**Figure 1:**
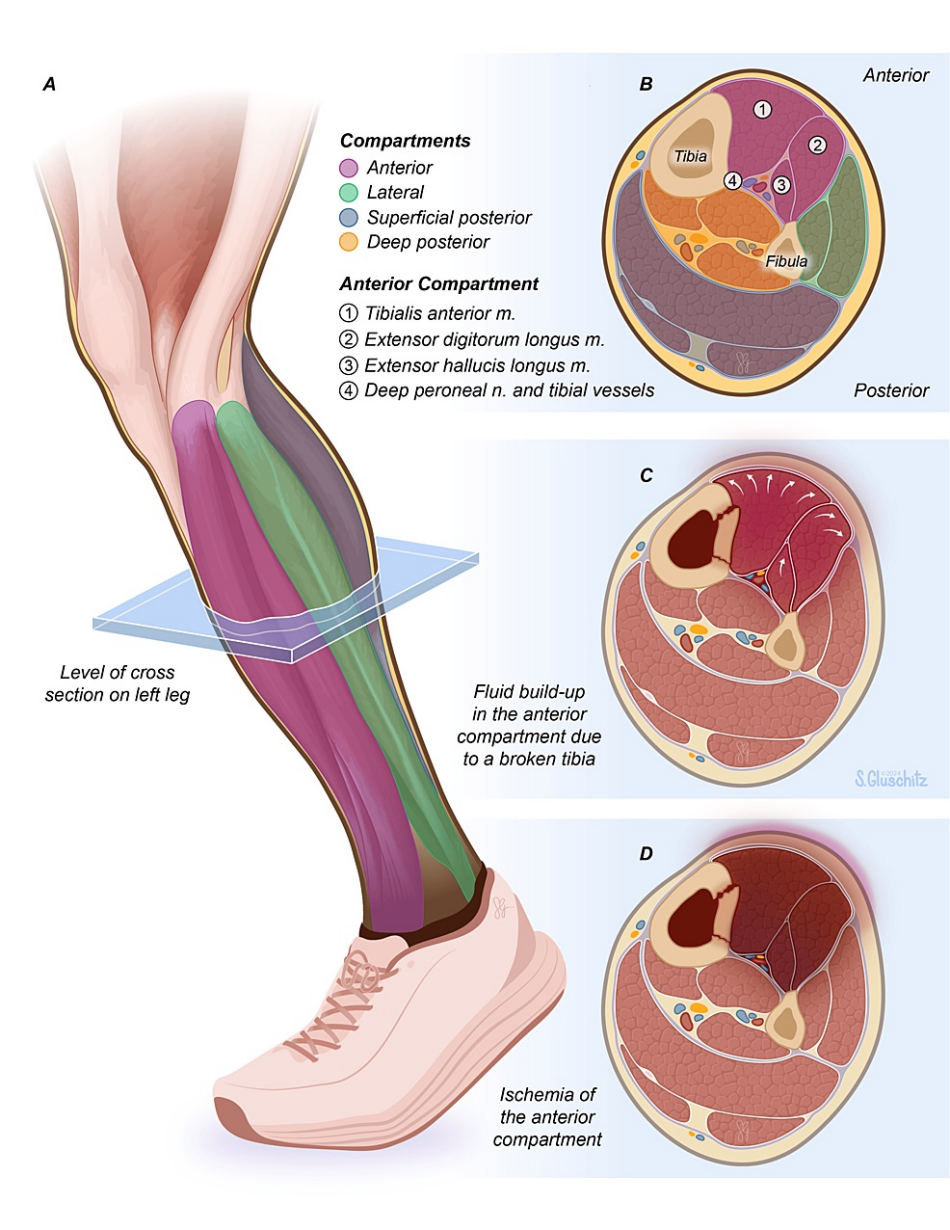
Acute Compartment Syndrome of the Lower Limb Figure 1a - Healthy lower limb cross-section of L4 dermatome. Figure 1b - Four compartments of a healthy lower limb: anterior, lateral, superficial posterior, and deep posterior. The anterior compartment is most commonly involved in ACS and contains muscles such as the tibialis anterior, extensor hallucis longus, extensor digitorum longus, and the deep peroneal nerve. Figure 1c - ACS developing following a tibial fracture. Fluid in the compartments increases pressure within the anterior compartment causing compression of vasculature and muscles. Figure 1d - Tissue ischemia and necrosis of the lower limb ensue when treatment is not obtained. Printed with permission from Sarah Gluschitz, MA, CMI © CC-BY-ND 2024.

The lower limb is divided into four different compartments, which include the anterior, lateral, superficial posterior, and deep posterior compartments. These compartments are separated by the interosseous membrane and intermuscular septa. The lateral compartment includes the perineal group of muscles responsible for plantarflexion and eversion of the foot, the posterior compartment contains the flexor group of muscles responsible for plantarflexion and inversion of the foot, and the anterior compartment contains the extensor group of muscles, which functions to primarily dorsiflex the foot and ankle [11]. ACS occurs most commonly in the anterior compartment of the lower leg largely as a result of fractures. The anterior compartment of the leg is more susceptible to ischemia because it is made up of red muscle fibers, which are highly dependent on aerobic metabolism [12]. Other common causes of anterior CS include trauma from crush injuries, gunshot wounds, surgical dressings, burns, and bleeding disorders to name a few [13]. The anterior compartment is innervated by the deep peroneal nerve and receives arterial supply from the tibial artery. The anterior compartment itself comprises the tibialis anterior, extensor hallucis longus, extensor digitorum longus, and fibularis tertius muscles. More specifically, the main functions of the muscles in this compartment are to dorsiflex the ankle, extend the toes, invert the foot via the tibialis anterior and flexor hallucis longus muscles, and evert the foot via the fibularis tertius muscle. When the pressure in the compartment exceeds the perfusion pressure of the vessel, this results in ACS, which can result in irreversible muscle and nerve damage and eventual muscle necrosis, as represented in Figure 1.

Pathophysiology

The body responds in many ways to the instigating trauma, leading to a cascade of events that eventually result in CS. ACS can be separated into two different etiologies: a reduction in the intracompartmental space or an increase in intracompartmental fluid volume. When the muscle and tissue are damaged, the pressure within the compartment rises. Hemodynamics between venous outflow and arterial outflow are disrupted when there is a rise in compartmental pressure [1]. This disrupts the microcirculation and tissue perfusion as the balance of capillary perfusion pressure and interstitial fluid pressure is affected [14,15]. Capillary permeability is increased, leading to fluid extravasation and raised interstitial fluid pressure [15]. When this fluid enters into a fixed compartment, there is an increase in the tissue and venous pressures [14]. These events compromise tissue perfusion, impairing oxygen and nutrient delivery to cells, resulting in cellular hypoxia. Hypoxia results from the shutdown of ATPase channels that maintain cellular osmotic balance [16]. The disruption of cell-membrane potential prompts the influx of chloride ions, leading to heightened cellular swelling, exacerbating the hypoxemic state, thus triggering a positive feedback loop [15]. As the cascade continues, tissue anoxia with eventual myonecrosis then proceeds [15]. Fibrosis of necrotic muscle tissue causes further functional impairment such as contractures [17]. The resultant ischemia also leads to a switch to anaerobic metabolism and the accumulation of metabolic byproducts such as lactate, indicating a metabolic ‘dead end’ [15].

The ischemic insult triggers an inflammatory response, resulting in the release of inflammatory mediators and cytokines. Research from 2018 studied the role of systemic inflammatory cytokine release in response to CS. This was evidenced by the upregulation of pro-inflammatory cytokines in the circulation such as TNF-α and IL-1β. Elevated intracompartmental pressures (ICPs) causing changes to microvascular perfusion and tissue injury all resulted in a significant increase in systemic TNF-α and IL-1β. The associated inflammation also resulted in increasing leukocyte count. TNF-α is a major pro-inflammatory cytokine released during trauma, infection, or during inflammation. It acts as a chemoattractant that brings neutrophils to the area, upregulates other cytokines, and ultimately activates cell death via apoptosis. Similarly, pro-inflammatory marker IL-1β is involved in cellular proliferation, differentiation, and apoptosis [18]. These factors contribute to a positive feedback loop, leading to edema that exacerbates the pressure elevation, further compromising blood flow and perpetuating tissue damage [19]. As pressures continue to rise, nerve structures within the compartment(s) are affected. Reduced blood flow and direct compression on nerves contribute to neurologic deficits, including sensory and motor disturbances. Paresthesia, numbness, and loss of function may ensue and typically develop after 30 minutes of ischemia with irreversible nerve damage beginning after 12-24 hours of total ischemia [20].

Furthermore, the ischemic insult does not stop there. When blood flow is restored, the reintroduction of oxygenated blood causes further tissue damage, or reperfusion injury, in what is called post-ischemic compartment syndrome [21,22]. This process is mediated by the interaction of oxygen-derived free radicals, which peroxidate the lipid component of cell membranes, leading to capillary permeability [23,24]. The subsequent release of toxic substances into systemic circulation as a result of reperfusion can lead to multiple organ damage and even death [25].

Diagnostic methods 

Timely and accurate diagnosis is crucial to prevent irreversible tissue damage. Clinical evaluation forms the cornerstone of diagnosing CS; however, various other diagnostic methods, including ICP measurements, aid in identifying CS in both adults and children [26]. Healthcare providers assess for classic signs and symptoms, including swelling with severe pain that is out of proportion to the injury, tense compartments upon palpation, pallor, paresthesia, pulselessness, and potential paralysis (AKA the five Ps) [18]. Diagnosing CS in non-verbal or preverbal children can be particularly challenging due to the reliance on subjective symptoms. Vigilance in recognizing early signs, such as agitation, anxiety, and increased analgesic requirements (AKA the three As), is essential in pediatric assessments [27]. These clinical findings, along with a thorough medical history and physical examination, raise suspicion for CS. Measurement of ICP is a recommended diagnostic tool to quantitate CS; however, the exact methodology of employing it in diagnosis is variable [28]. In both children and adults, interpreting ICPs can be challenging due to variations based on age, anatomical differences, and compliance during measurements.

Invasive Methods

The pressure of a normal myofascial compartment is quoted to be less than 10 mmHg [19]. An ICP of at least 30 mmHg was selected as an indication for fasciotomy by some authors because this is the threshold where capillary pressure fails to maintain blood flow to muscles according to Starling’s forces [29,30]. In contrast, other authors have based their numerical pressure threshold on when ICPs increase to within 30 mmHg of the diastolic blood pressure [26,31,32]. Popular instruments for measuring ICP include the Stryker Quick Pressure Monitor Instrument, the manometric intravenous (IV) pump method, and the Whitesides Infusion Technique. The Stryker monitor is a handheld device that is easy to use and accurate at determining ICPs with the advantage of not requiring complex equipment for operation [33,34]. It involves simply placing a needle in the compartment of interest, zeroing the instrument, injecting a saline solution into the device, and reading the measurements [35]. Due to possible user error, multiple measurements may need to be taken, with the final measurement being determined from the average. The process is repeated for any additional compartments of interest. The IV pump method is used similarly by using an IV pump that has pressure sensing capability [33]. This method has also been found to yield accurate and reliable results. Because the Stryker instrument is expensive, some hospitals may not have access to them. In this case, the IV pump method is a reliable backup, as they are readily available in all hospitals [33]. The Whitesides technique is more intricate and involves the usage of two plastic extension tubes, two 18-gauge needles, one 20 mL syringe, one three-way stopcock, one vial of normal saline, and one mercury manometer [36]. Unfortunately, the Whitesides method has yielded unreliable results in multiple studies and is not highly recommended [33,37]. Two other similar methods of determining ICPs include the Slit Catheter and Wick Catheter methods. In one study comparing the two, the Slit Catheter method was proven to be cheaper, has less time to construct, and was suitable for long-term pressure monitoring [38].

Noninvasive Methods

In addition, there are several other less invasive diagnostic aids that may also be used. Imaging studies such as compartment pressure monitoring devices, near-infrared spectroscopy (NIRS), Laser Doppler Flowmetry, and ultrasonic devices are emerging as adjuncts to aid in recognizing CS. These techniques provide continuous monitoring of ICPs and tissue oxygenation, offering valuable information for decision-making in adults and may particularly be useful in pediatric patients [19]. NIRS and Laser Doppler Flowmetry may have more utility in CECS. NIRS tissue monitors utilize the distinct absorption properties of light to offer a noninvasive, continuous method for measuring the percentage of oxygen-saturated hemoglobin in tissue located 2-3 cm below the skin [39,40]. One study elucidated that NIRS may have diagnostic potential for CS; however, it highlighted constraints arising from confounding factors such as body habitus and the anatomical placement of sensor pads, which could influence outcomes [41]. Laser Doppler flowmetry (LDF) is a non-invasive technique in which microcirculation changes in tissue are studied [42]. One study wanted to assess the use of LDF in diagnosing CECS because ICP does not account for the vascular state. Their study revealed significant changes in muscle blood flow over time, which differed between control subjects and those with CECS [43]. Additionally, ultrasonic devices have also been shown to provide significant data in the diagnosis of CS. It involves the usage of pulsed phase-locked loop ultrasonic devices to measure sub-micrometer displacements of the fascia wall [44,45]. Another study used ultrasonic devices to correlate perfusion pressure, in addition to fascial displacement, as another way to aid in the diagnosis of CS [46].

The American Academy of Orthopaedic Surgeons provides an evidence-based clinical practice guideline in the diagnosis and management of ACS. They illustrated that, although biomarkers provide limited and moderate evidence of CS, they are not specific to the diagnosis [47]. These biomarkers include myoglobinuria, serum troponin, and lactate concentration [47]. A different study also evaluated creatine kinase, fatty acid-binding protein (FABP), and white blood cell count as other means of diagnosis, but found these to be nonspecific as well [34].

We would also like to take time to discuss a newly developed, FDA-approved, minimally invasive device called the MYO1 that is capable of continuously measuring variations in ICPs. A study comparing the MYO1 to other devices of similar use revealed that the MYO1 device provided the truest indicator of reference pressure [48]. A case report describes the device as an indwelling sensor inserted into a muscle compartment that gives results on a display tethered to the compartment in question. It can also feed directly to the care provider through a cell phone application [49]. This was the first case report describing the feasibility and safety of this new monitoring device and aimed to highlight two important clinical implications of its use: (1) avoiding unnecessary fasciotomies and (2) alerting caregivers to ACS before clinical signs presented [50].

Management

Prompt intervention to prevent irreversible tissue damage and limb loss is necessary to treat CS, especially ACS. The primary treatment modality for CS involves surgical intervention, although approaches may differ based on the clinical scenario, patient age, and severity of the condition. The cornerstone of treatment for ACS, in both adult and pediatric patients, involves immediate surgical decompression via fasciotomy [28]. This procedure entails making incisions through the affected compartment's fascia, relieving pressure, and restoring blood flow to alleviate tissue ischemia. Fasciotomy is essential in both adults and children to prevent irreversible tissue damage. Prior to fasciotomy removal of any external dressings, such as casts, compressive bandages or plaster is recommended. One study investigated the effect of removing compressive external dressings on reducing ICP in dogs. It was found that just by cutting the plaster alone reduced ICP by a mean of 65% [50]. Although this study resulted in a significant decrease in ICP, the authors concluded that even after the removal of compressing external dressings, continued evaluation for CS is necessary, and fasciotomy may still be required [50].

The timing of surgery is also of utmost importance. Prompt surgical intervention is critical. Delays in treatment may lead to irreversible tissue damage and complications such as needless loss of function and possible amputation of the involved limb [26]. Therefore, surgical decompression should be undertaken as soon as CS is suspected or diagnosed [51]. The choice of incision technique and extent of the fasciotomy depend on the specific compartment(s) involved [51]. In the lower limb, decompression can be performed by either a single lateral incision or combined anterolateral and posteromedial incisions [52]. Either of these incisions is recommended to be a minimum of 15 cm [53]. The double incision method can reduce soft tissue support, so in patients with tibial fractures, for example, the single incision method is recommended to sustain the stability of the fracture [54]. For ACS presenting in the thigh, a single lateral incision is typically utilized in comparison with two incisions as it allows adequate decompression of all three compartments; however, two incisions may prevent muscle herniation [20,55]. In cases that present in the foot, the choice of incision lies in which compartment(s) ACS is presenting. For decompression of the forefoot, two dorsal incisions over the second and fourth metatarsals are used [56]. For decompression of the calcaneal, medial, and superficial compartments, a medial incision is used [51]. Lastly, a lateral incision beginning at the lateral malleolus extending to the forefoot between the fourth and fifth metatarsals is used to decompress the lateral compartment [19,51,57]. Additionally, one study found that, in regards to infants and toddlers, the diagnosis of CS was delayed by up to 24 hours after injury [58]. However, despite this delay, outcomes after fasciotomy still remained favorable even up to 48-72 hours after injury [58]. In patients with CECS, the mainstay treatment is also the traditional open fasciotomy; however, nonoperative management strategies are readily being introduced [59]. Open fasciotomy is the treatment of choice due to increased success rates, increased patient satisfaction, and favorable functional outcomes as compared to nonoperative treatments [60,61]. However, these results are multifactorial and may be due to factors such as patient age, location of the compartment(s) affected, fascia thickness, duration of symptoms, and compartment pressure measurements [59].

Advancements in surgical approaches have introduced minimally invasive techniques, such as endoscopic, minimal-incision, and ultrasound-guided fasciotomy, aimed at achieving decompression while minimizing soft tissue trauma [59]. One study found that endoscopic approaches yielded positive outcomes for the release of the anterior and lateral compartments but cautioned against the usage of deep posterior compartment release due to the risk of hemorrhage [62]. Studies of the single minimal-incision and ultrasound-guided fasciotomies have been performed with successful results; however, more research is needed to understand the true incidence of complications, success rates, and generalizability of these methods [59].

Following fasciotomy, meticulous wound care and monitoring for signs of infection are essential to prevent complications. Primary closure of the wound is not commonly performed, and, in most cases, it is often not even possible. Delayed closure with tissue approximation or via applied skin grafts over the healthy granulation tissue finalizes the repair phase [19,20,63]. Regular assessments of the open wound must be managed with strict sterility and wound care protocol [63]. The usage of vacuum-assisted closure (VAC) or shoelace suturing techniques may also be employed [20,64]. Challenges of fasciotomy include superficial peroneal nerve injury, saphenous vein and nerve injury, incomplete release of compartment pressure, as well as complications in wound closure and skin grafting [65].

In addition to wound care and neurovascular monitoring, early initiation of rehabilitation and physiotherapy is essential for optimizing functional recovery post-surgery. These interventions aid in restoring strength, range of motion, and functional capacity of the affected limb [63]. One study emphasized the importance of initiating the 'PRICE' (Protection, Resting, Ice, Compression, and Elevation) rehabilitation steps, followed by gradual progression to re-establishing range of motion and soft tissue mobility through stretching, neurodynamic mobilizations, strengthening, and finally, incorporation of biomechanical analysis during activity [66]. Although there is a standard rehabilitation protocol described in the literature, more research is needed on postoperative rehabilitation outcomes to contribute to the development of better programs in the future [62]. Successful management of CS through timely fasciotomy generally yields favorable outcomes with reduced long-term complications. Complications such as infection, nerve damage, muscle contractures, or chronic pain may occur, necessitating ongoing monitoring and management [17]. 

Discussion 

Surgical decompression via fasciotomy remains the gold standard of care for both adults and children with CS [28]. Prompt intervention is necessary to prevent delays, which may lead to irreversible tissue damage and necrosis [26]. The mode of injury, clinical scenario, patient age, and severity are all factors considered before intervention. Single- or dual-incision fasciotomy is the treatment of choice, allowing for vital decompression of the compartment being compromised. The single-incision technique gives access to the lateral or posterolateral aspect of the lower limb, while dual-incision involves the anterolateral aspect of the lower limb, allowing for decompression [65]. In both single- and dual-incision approaches, after appropriate debridement of nonviable muscle has been performed, the postoperative protocol includes dressing the wound with a VAC device [65].

A retrospective analysis studied patients who had undergone tibia and/or fibula fractures with and without compartment syndrome. The risk factors for fasciotomy and related complications were studied. With the 98 patients who underwent fasciotomy compared to the 1,686 who did not, significant differences were found in terms of age, sex, post-surgical complications, duration of operation, and post-operative care [67]. Similarly, early vs. late fasciotomy was studied in patients with extreme vascular injuries and their outcomes. The 612 patients were divided into two groups: those who had fasciotomies in a time frame of less than eight hours and those who had undergone fasciotomy after eight hours [68]. A total of 543 patients in the early group had a lower rate of iliac artery injury compared to the late fasciotomy group (5.9% vs. 23.2%, P<0.001). Early fasciotomy patients also had a lower amputation rate (8.5% vs 24.6%, P<0.001) and a lower infection rate (6.6% vs. 14.5%, P=0.28) [68]. The duration of hospital stay and post-surgical outcomes were lower in the early fasciotomy group compared to the late group as well (18.5 ± 20.7 days vs. 24.2 ± 14.7 days, P=0.007) [68].

Though highly effective for rapid decompression and prevention of tissue ischemia and necrosis, fasciotomy includes a host of complications including increased hospital stay, wound infections, need for further surgery/skin grafting, delayed bone healing, and not limited to nerve injury. Risks can be mitigated with proper wound closure [69]. Though surgical intervention is the treatment of choice and has proven to have successful outcomes, advancements using minimally invasive techniques such as endoscopic, minimal-incision, and ultrasound-guided fasciotomy techniques are still to be further studied but have also proven successful [59]. Studies conducted by Grechenig et al. studied minimally invasive fasciotomy techniques and the risk of iatrogenic injury in 60 lower extremity cadavers. Complete compartment release was achieved in 97%-100% of the cadavers with no injury to any vessel or superficial peroneal nerve [70]. This study provides evidence that a more noninvasive approach may be beneficial to patients in the long run and has the potential to become the future of CS treatment.

Limitations 

This review, however, has several limitations. Firstly, the narrative format lacks a systematic approach, potentially leading to selection bias and the exclusion of relevant studies. This may affect the comprehensiveness of the analysis regarding various diagnostic and treatment modalities, thus limiting the generalizability of the findings. Secondly, the review predominantly focuses on surgical and immediate post-surgical interventions, with an insufficient exploration of long-term outcomes and non-surgical management strategies. Lastly, there is a lack of representation of diverse patient populations, which may impact the applicability of the findings across different demographic groups. Future research should consider a systematic review or meta-analysis to provide more robust evidence and address these limitations.

## Conclusions

In conclusion, CS represents a critical condition requiring prompt recognition and immediate intervention to prevent irreversible tissue damage and limb loss. This narrative review highlights the importance of understanding the intricate anatomy and pathophysiology underlying CS, as well as the significance of timely diagnosis and effective management strategies. Surgical decompression via fasciotomy remains the gold standard treatment for acute cases, emphasizing the necessity for rapid surgical response to mitigate complications such as muscle necrosis and nerve damage. Advances in diagnostic tools and minimally invasive surgical techniques show promise in improving outcomes and reducing complications associated with traditional methods. Ongoing research and development in early diagnostic methods and innovative treatment approaches are crucial to enhance the management and prognosis of patients with both acute and chronic compartment syndrome.

## References

[1] Torlincasi AM, Lopez RA, Waseem M (2024). Acute compartment syndrome. StatPearls.

[2] Shore BJ, Glotzbecker MP, Zurakowski D, Gelbard E, Hedequist DJ, Matheney TH (2013). Acute compartment syndrome in children and teenagers with tibial shaft fractures: incidence and multivariable risk factors. J Orthop Trauma.

[3] Hak DJ (2019). Acute compartment syndrome in childrenors. Compartment Syndrome.

[4] Janzing HM (2007). Epidemiology, etiology, pathophysiology and diagnosis of the acute compartment syndrome of the extremity. Eur J Trauma Emerg Surg.

[5] Brennan FH Jr, Kane SF (2003). Diagnosis, treatment options, and rehabilitation of chronic lower leg exertional compartment syndrome. Curr Sports Med Rep.

[6] Vajapey S, Miller TL (2017). Evaluation, diagnosis, and treatment of chronic exertional compartment syndrome: a review of current literature. Phys Sportsmed.

[7] Patterson Tichy AM, Bradley C (2019). Unilateral exertional compartment syndrome in a pediatric competitive figure skater. Cureus.

[8] Touliopolous S, Hershman EB (1999). Lower leg pain. Diagnosis and treatment of compartment syndromes and other pain syndromes of the leg. Sports Med.

[9] Hurschler C, Vanderby R Jr, Martinez DA, Vailas AC, Turnipseed WD (1994). Mechanical and biochemical analyses of tibial compartment fascia in chronic compartment syndrome. Ann Biomed Eng.

[10] Rajasekaran S, Kvinlaug K, Finnoff JT (2012). Exertional leg pain in the athlete. PM R.

[11] Mostafa E, Graefe SB, Varacallo M (2024). Anatomy, bony pelvis and lower limb: leg posterior compartment. StatPearls.

[12] Taylor RM, Sullivan MP, Mehta S (2012). Acute compartment syndrome: obtaining diagnosis, providing treatment, and minimizing medicolegal risk. Curr Rev Musculoskelet Med.

[13] Khan IA, Mahabadi N, D’Abarno A, Varacallo M (2023). Anatomy, bony pelvis and lower limb: leg lateral compartment. StatPearls.

[14] Donaldson J, Haddad B, Khan WS (2014). The pathophysiology, diagnosis and current management of acute compartment syndrome. Open Orthop J.

[15] Merle G, Harvey EJ (2019). Pathophysiology of compartment syndrome. Compartment Syndrome.

[16] Von Keudell AG, Weaver MJ, Appleton PT (2015). Diagnosis and treatment of acute extremity compartment syndrome. Lancet.

[17] Santi MD, Botte MJ (1995). Volkmann's ischemic contracture of the foot and ankle: evaluation and treatment of established deformity. Foot Ankle Int.

[18] Donohoe E, Bihari A, Schemitsch E, Sanders D, Lawendy AR (2018). Compartment syndrome-induced muscle injury is diminished by the neutralization of pro-inflammatory cytokines. OTA Int.

[19] Mabvuure NT, Malahias M, Hindocha S (2012). Acute compartment syndrome of the limbs: current concepts and management. Open Orthop J.

[20] Gourgiotis S, Villias C, Germanos S, Foukas A, Ridolfini MP (2007). Acute limb compartment syndrome: a review. J Surg Educ.

[21] Perry MO (1988). Compartment syndromes and reperfusion injury. Surg Clin North Am.

[22] Beyersdorf F (1991). Protection of the ischemic skeletal muscle. Thorac Cardiovasc Surg.

[23] Grace PA (1994). Ischaemia-reperfusion injury. Br J Surg.

[24] Durán WN, Takenaka H, Hobson RW 2nd (1998). Microvascular pathophysiology of skeletal muscle ischemia-reperfusion. Semin Vasc Surg.

[25] Blaisdell FW (2002). The pathophysiology of skeletal muscle ischemia and the reperfusion syndrome: a review. Cardiovasc Surg.

[26] Whitesides TE, Haney TC, Morimoto K, Harada H (1975). Tissue pressure measurements as a determinant for the need of fasciotomy. Clin Orthop Relat Res.

[27] Frei B, Sommer-Joergensen V, Holland-Cunz S, Mayr J (2020). Acute compartment syndrome in children; beware of "silent" compartment syndrome: a CARE-compliant case report. Medicine (Baltimore).

[28] Lin JS, Samora JB (2020). Pediatric acute compartment syndrome: a systematic review and meta-analysis. J Pediatr Orthop B.

[29] Mubarak SJ, Owen CA, Hargens AR, Garetto LP, Akeson WH (1978). Acute compartment syndromes: diagnosis and treatment with the aid of the wick catheter. J Bone Joint Surg Am.

[30] Hargens AR, Akeson WH, Mubarak SJ (1978). Fluid balance within the canine anterolateral compartment and its relationship to compartment syndromes. J Bone Joint Surg Am.

[31] Kanj WW, Gunderson MA, Carrigan RB, Sankar WN (2013). Acute compartment syndrome of the upper extremity in children: diagnosis, management, and outcomes. J Child Orthop.

[32] McQueen MM, Court-Brown CM (1996). Compartment monitoring in tibial fractures. The pressure threshold for decompression. J Bone Joint Surg Br.

[33] Uliasz A, Ishida JT, Fleming JK, Yamamoto LG (2003). Comparing the methods of measuring compartment pressures in acute compartment syndrome. Am J Emerg Med.

[34] Shadgan B, Menon M, O'Brien PJ, Reid WD (2008). Diagnostic techniques in acute compartment syndrome of the leg. J Orthop Trauma.

[35] Halanski MA, Morris MR, Lee Harper B, Doro C (2015). Intracompartmental pressure monitoring using a handheld pressure monitoring system. JBJS Essent Surg Tech.

[36] Whitesides TE Jr, Haney TC, Harada H, Holmes HE, Morimoto K (1975). A simple method for tissue pressure determination. Arch Surg.

[37] Boody AR, Wongworawat MD (2005). Accuracy in the measurement of compartment pressures: a comparison of three commonly used devices. J Bone Joint Surg Am.

[38] Shakespeare DT, Henderson NJ, Clough G (1982). The slit catheter: a comparison with the wick catheter in the measurement of compartment pressure. Injury.

[39] Shuler MS, Reisman WM, Whitesides TE Jr, Kinsey TL, Hammerberg EM, Davila MG, Moore TJ (2009). Near-infrared spectroscopy in lower extremity trauma. J Bone Joint Surg Am.

[40] Arimoto H, Egawa M, Yamada Y (2005). Depth profile of diffuse reflectance near-infrared spectroscopy for measurement of water content in skin. Skin Res Technol.

[41] Cole AL, Herman RA Jr, Heimlich JB, Ahsan S, Freedman BA, Shuler MS (2012). Ability of near infrared spectroscopy to measure oxygenation in isolated upper extremity muscle compartments. J Hand Surg Am.

[42] Micheels J, Alsbjorn B, Sorensen B (1984). Laser doppler flowmetry. A new non-invasive measurement of microcirculation in intensive care?. Resuscitation.

[43] Abraham P, Leftheriotis G, Saumet JL (1998). Laser Doppler flowmetry in the diagnosis of chronic compartment syndrome. J Bone Joint Surg Br.

[44] Lynch JE, Heyman JS, Hargens AR (2004). Ultrasonic device for the noninvasive diagnosis of compartment syndrome. Physiol Meas.

[45] Wiemann JM, Ueno T, Leek BT, Yost WT, Schwartz AK, Hargens AR (2006). Noninvasive measurements of intramuscular pressure using pulsed phase-locked loop ultrasound for detecting compartment syndromes: a preliminary report. J Orthop Trauma.

[46] Garabekyan T, Murphey GC, Macias BR, Lynch JE, Hargens AR (2009). New noninvasive ultrasound technique for monitoring perfusion pressure in a porcine model of acute compartment syndrome. J Orthop Trauma.

[47] American Academy of Orthopaedic Surgeons (2024). Management of acute compartment syndrome evidence-based clinical practice guideline. Management of Acute Compartment Syndrome Evidence- Based Clinical Practice Guideline. aaos.org/acscpg Published December.

[48] Merle G, Comeau-Gauthier M, Tayari V (2020). Comparison of three devices to measure pressure for acute compartment syndrome. Mil Med.

[49] Montreuil J, Corban J, Reindl R, Harvey EJ, Bernstein M (2022). Novel digital continuous sensor for monitoring of compartment pressure: a case report. OTA Int.

[50] Garfin SR, Mubarak SJ, Evans KL, Hargens AR, Akeson WH (1981). Quantification of intracompartmental pressure and volume under plaster casts. J Bone Joint Surg Am.

[51] Frink M, Hildebrand F, Krettek C, Brand J, Hankemeier S (2010). Compartment syndrome of the lower leg and foot. Clin Orthop Relat Res.

[52] Cooper GG (1992). A method of single-incision, four compartment fasciotomy of the leg. Eur J Vasc Surg.

[53] Malik AA, Khan WS, Chaudhry A, Ihsan M, Cullen NP (2009). Acute compartment syndrome--a life and limb threatening surgical emergency. J Perioper Pract.

[54] DeLee JC, Stiehl JB (1981). Open tibia fracture with compartment syndrome. Clin Orthop Relat Res.

[55] Ojike NI, Roberts CS, Giannoudis PV (2010). Compartment syndrome of the thigh: a systematic review. Injury.

[56] Myerson MS (1988). Experimental decompression of the fascial compartments of the foot--the basis for fasciotomy in acute compartment syndromes. Foot Ankle.

[57] Echtermeyer V, Muhr G, Oestern HJ, Tscherne H (1982). [Surgical treatment of compartmental syndromes (author's transl)]. Unfallheilkunde.

[58] Broom A, Schur MD, Arkader A, Flynn J, Gornitzky A, Choi PD (2016). Compartment syndrome in infants and toddlers. J Child Orthop.

[59] Buerba RA, Fretes NF, Devana SK, Beck JJ (2019). Chronic exertional compartment syndrome: current management strategies. Open Access J Sports Med.

[60] Fraipont MJ, Adamson GJ (2003). Chronic exertional compartment syndrome. J Am Acad Orthop Surg.

[61] Pasic N, Bryant D, Willits K, Whitehead D (2015). Assessing outcomes in individuals undergoing fasciotomy for chronic exertional compartment syndrome of the leg. Arthroscopy.

[62] Lohrer H, Nauck T (2007). Endoscopically assisted release for exertional compartment syndromes of the lower leg. Arch Orthop Trauma Surg.

[63] Altan L (2023). Postoperative rehabilitation of compartment syndrome following fasciotomy. Turk J Phys Med Rehabil.

[64] Zannis J, Angobaldo J, Marks M, DeFranzo A, David L, Molnar J, Argenta L (2009). Comparison of fasciotomy wound closures using traditional dressing changes and the vacuum-assisted closure device. Ann Plast Surg.

[65] Singh K, Bible JE, Mir HR (2015). Single and dual-incision fasciotomy of the lower leg. JBJS Essent Surg Tech.

[66] Schubert AG (2011). Exertional compartment syndrome: review of the literature and proposed rehabilitation guidelines following surgical release. Int J Sports Phys Ther.

[67] Mittlmeier AS, Pape HC, Neuhaus V, Canal C (2024). The impact of fasciotomy on inpatient outcomes in lower leg fracture management. Eur J Orthop Surg Traumatol.

[68] Farber A, Tan TW, Hamburg NM (2012). Early fasciotomy in patients with extremity vascular injury is associated with decreased risk of adverse limb outcomes: a review of the National Trauma Data Bank. Injury.

[69] Igoumenou VG, Kokkalis ZT, Mavrogenis AF (2019). Fasciotomy wound management. Compartment Syndrome.

[70] Grechenig P, Valsamis EM, Müller T, Gänsslen A, Hohenberger G (2020). Minimally invasive lower leg fasciotomy for chronic exertional compartment syndrome-how safe is it? A cadaveric study. Orthop J Sports Med.

